# Human Immunodeficiency Virus: A Dark Cloud With Silver Lining During the COVID-19 Pandemic

**DOI:** 10.7759/cureus.9302

**Published:** 2020-07-20

**Authors:** Rama Kanth Pata, Abolfazl Ahmady, Roudabeh Kiani

**Affiliations:** 1 Internal Medicine, Interfaith Medical Center, Brooklyn, USA; 2 Pulmonary Medicine, Interfaith Medical Center, Brooklyn, USA

**Keywords:** hiv, covid-19, sars-cov-2, immune deficiency, remdesavir

## Abstract

In December 2019, China reported a cluster of pneumonia patients infected by a new virus from the coronavirus family called severe acute respiratory syndrome coronavirus 2 (SARS-CoV-2). The virus quickly spread around the world and infected millions of people, and the World Health Organization (WHO) declared coronavirus disease 2019 (COVID-19) a pandemic on March 11, 2020. Although some patients show only mild or even asymptomatic response to this infection, severe disease with rapid progression to acute respiratory distress and multiorgan failure is also commonly seen. In this report, we discuss three cases of HIV patients who survived COVID-19.

## Introduction

Severe acute respiratory syndrome coronavirus 2 (SARS-CoV-2) is a coronavirus with a positive-sense single-stranded ribonucleic acid (RNA). It is believed to have originated in bats because of the 96% genomic similarities it has with bat coronaviruses. The high incubation period (2-14 days) in conjunction with the high survivability of this virus and a high reproductive number (R0 ranging from 1.4 to 6.49, with a mean of 3.28 in one study and 2 to 3.5 in another) [1-3] have caused it to spread quickly throughout the world. On March 11, 2020, the World Health Organization (WHO) declared the coronavirus disease 2019 (COVID-19) outbreak a global pandemic. The COVID-19 pandemic has expanded rapidly worldwide. The number of cases has skyrocketed as days passed, and mortality has rapidly increased globally. In this report, we present three cases of HIV patients who survived COVID-19.

## Case presentation

Case 1

On March 22, 2020, a 67-year-old female with a past medical history of asthma, coronary artery disease (status post-coronary artery bypass graft two years ago), hypertension, hyperlipidemia, and HIV on antiretroviral medications [bictegrav/emtricit/tenofov ala (Biktarvy® 50-200-25 mg tablet, Gilead Sciences, Foster City, CA) and darunavir/cobicistat (Prezcobix® 800 mg-150 mg tablet, Janssen Pharmaceutica, Beerse, Belgium)] was brought in by emergency medical services (EMS) for progressively worsening shortening of breath associated with weakness and two episodes of watery non-bloody diarrhea for one day. She had sought medical attention two days ago at an emergency department where she had been tested for COVID-19 [reverse transcription-polymerase chain reaction (RT-PCR)]. She had been discharged on levofloxacin. She returned to the hospital for worsening of symptoms but denied any new symptoms including fever or cough. The COVID-19 RT-PCR came back positive later. Her chest CT scan showed multifocal patchy consolidations of the bilateral upper and lower lobes, and the electrocardiogram showed normal sinus rhythm with corrected QT interval (QTc) of 453 ms and T wave inversion in V2. Two sets of blood and urine cultures were negative. Other laboratory findings are presented in Table 1 (column: case 1).

**Table 1:** Blood lab results of the three cases WBC: white blood count; Hgb: hemoglobin; ESR: erythrocyte sedimentation rate; PT: prothrombin time; INR: international normalized ratio; APPT: activated partial thromboplastin time; CRP: C-reactive protein; Cr: creatinine; HIV: human immunodeficiency virus; RNA: ribonucleic acid; PCR: polymerase chain reaction

Lab tests	Case 1	Case 2	Case 3	Units
WBC	5,100	9,300	6,300	/uL
Hgb	12.4	9.3	12.9	g/dL
Platelet	207,000	324,000	448,000	/uL
ESR	110	>120	67	Mm/hr
PT	18	11.9	15.9	Sec
INR	1.54	1.02	1.36	----
APTT	31.3	32.3	33.4	Sec
D-dimer (max)	2,631 (18,000)	1,436 (3,849)	4,491 (5,796)	ng/ml
Fibrinogen	801	482	NA	mg/dL
Fibrinogen antigen	618	NA	NA	mg/dL
Lactic acid	1.5	NA	0.8	mmol/L
Lactate dehydrogenase	725	956	905	U/L
Troponin I	0.04	0.04	0.00	ng/mL
CRP	184	341	NA	mg/L
Ferritin	NA	NA	5,045	ng/mL
Cr	1.64	16.9	1.23	mg/dL
Procalcitonin	NA	30.70	0.07	ng/mL
Absolute CD4 count	157	41	307	Cells/uL
Absolute CD8 count	248	72	220	Cells/uL
CD4/CD8	0.63	0.57	1.4	
Lymphocyte count	1,800	1,200	6,300	/uL
HIV-1 RNA (PCR)	<20	35	<20	

**Figure 1:**
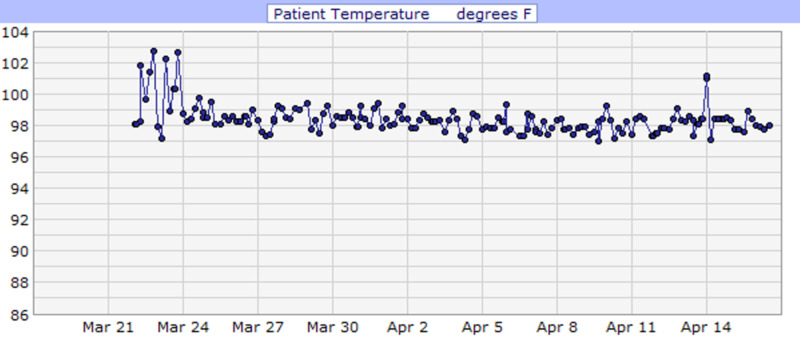
Case 1 temperature during admission (°F)

**Figure 2:**
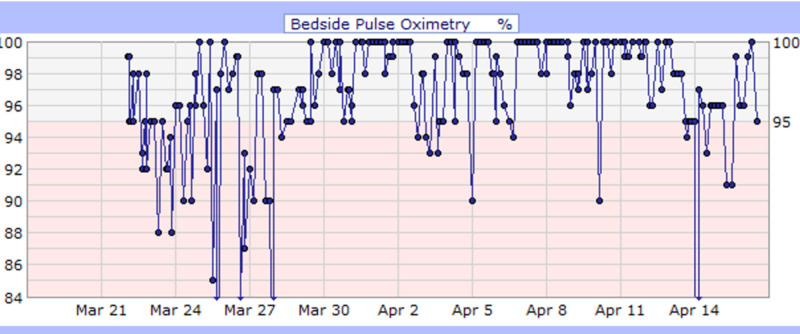
Case 1 bedside pulse oximetry (%)

She was admitted for COVID-19 pneumonia and placed on cardiac monitoring due to elevated troponin levels (0.04 ng/ml) and d-dimer (2,631 ng/ml). Additionally, she was started on IV antibiotics (ceftriaxone and azithromycin) and IV fluids. CT scan of the chest on admission incidentally showed cholelithiasis without cholecystitis likely due to acute pancreatitis; amylase and lipase were 1,562 U/L and >900 U/L respectively, and toxicology was negative for alcohol. She was kept NPO; a hepatobiliary iminodiacetic acid (HIDA) scan was performed after she refused a CT scan of the abdomen, which showed no scintigraphic evidence of biliary obstruction. In addition, heparin [5,000 units every 12 hours (Q12H)] was started as standard deep vein thrombosis (DVT) prophylaxis. She had an acute drop in hemoglobin during her stay, and a fecal occult blood test was performed, which came back positive (March 23, 2020). Therefore, heparin was discontinued, and she remained NPO in view of concomitant pancreatitis and was started on an IV proton pump inhibitor. Hemoglobin levels were monitored, and no further drop in hemoglobin level, hematemesis, or melena was observed. Her clinical condition improved, hence diet was modified based on what she could tolerate. During the first four days of admission, she had fluctuating fever spikes (Figure 1). Additionally, throughout her stay, she had a few episodes of drops in her oxygen saturation (Figure 2). Consequently, she was placed on high flow oxygen cannula (2.0 L/min), and her oxygen saturation subsequently improved (Table 2). She completed a course of hydroxychloroquine (400 mg daily PO for three days followed by 200 mg PO Q12H for three days), azithromycin [500 mg daily intravenous piggyback (IVPB) for 10 days] and ceftriaxone (1 gm daily IVPB for 10 days) while QTc interval and electrolytes level were monitored. Her troponin levels declined, and her clinical condition improved significantly with medical management and nursing care. Consequently, she was discharged on April 16, 2020, with instructions on social distancing and appointments to follow up.

**Table 2:** Case 1 arterial blood gas from April 9 to April 14, 2020 pCO_2_: partial pressure of carbon dioxide; pO_2_: partial pressure of oxygen; FiO_2_: fraction of inspired oxygen; P/F: PaO_2_/FiO_2_

Parameters	April 9, 2020	April 14, 2020
PH	7.295	7.462
pCO_2_	38.3	37.6
pO_2_	50.7	68.2
O_2_ saturation	86.4%	93.6%
O_2_ delivery	Room air	Nasal cannula
FiO_2_	21%	28% (2.0 L nasal cannula)
P/F ratio	241	243

Case 2

**Figure 3:**
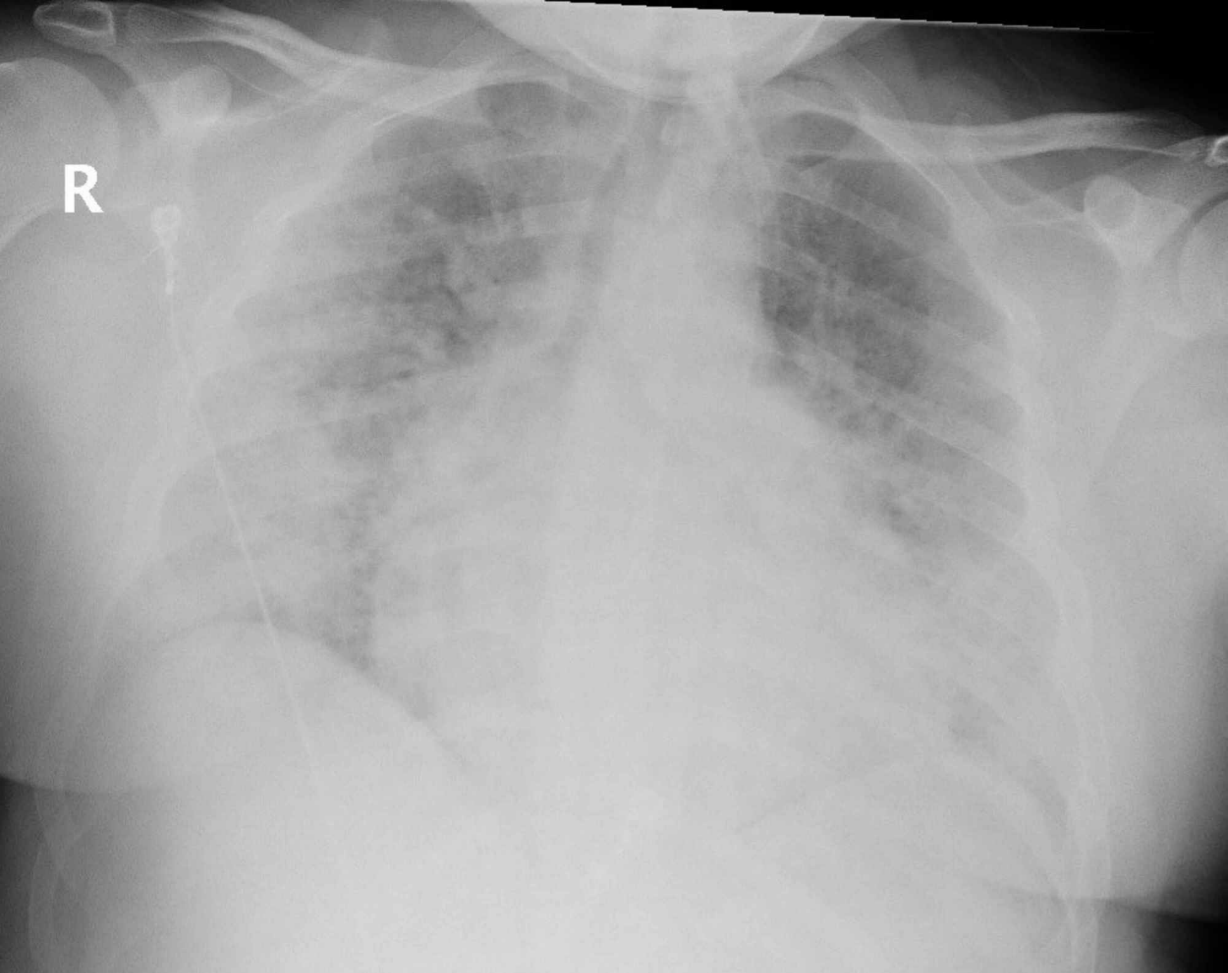
Case 2 chest X-ray on March 26, 2020

On March 26, 2020, a 31-year-old male with a past medical history of end-stage renal disease on hemodialysis (Tuesday, Thursday, and Saturday) and HIV on antiretroviral medications [darunavir ethanolate (Prezista®, Janssen Pharmaceutica, Beerse, Belgium), dolutegravir/rilpivirine (Juluca 50-25 mg tablet, ViiV Healthcare Limited, The Research Triangle, NC), and ritonavir] presented to the emergency department for dry cough and shortness of breath for five days along with constant central abdominal pain for two days. He denied any fever, nausea, or vomiting. His chest X-ray showed extensive bilateral patchy alveolar density in both lungs (Figure 3), and his COVID-19 test (RT-PCR) came back positive. Blood test results are presented in Table 1 (column: case 2).

**Figure 4:**
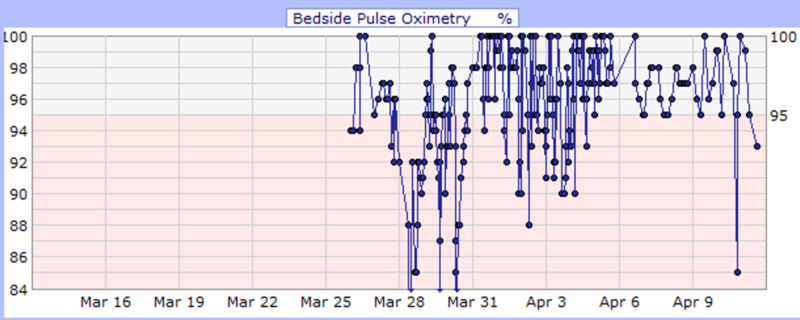
Case 2 bedside pulse oximetry (%)

**Figure 5:**
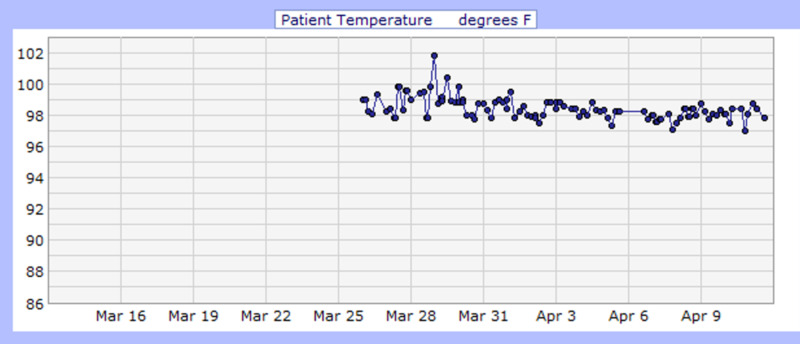
Case 2 temperature (°F)

He developed desaturation with increasing respiratory distress and altered mental status during his medical floor stay. Arterial blood gas (ABG) at 4 L nasal cannula showed hypoxia with PaO_2_ of 47 mmHg and O_2_ saturation of 78.5% (Figure 4). Consequently, he was upgraded to the intensive care unit due to the impeding respiratory arrest for close monitoring and further management; however, he refused intubation. During his stay, he began saturating well on a non-rebreather (Table 3) and was downgraded to the floor. He finished a course of ceftriaxone (1 gm daily IVPB for eight days) and azithromycin (500 mg IVPB daily for seven days) and received hydroxychloroquine (400 mg PO daily) for 10 days while QTc interval and electrolytes level were monitored. Additionally, he received hemodialysis as per his schedule. On April 3, 2020, sputum culture grew Klebsiella pneumonia for which he received antibiotics (meropenem 500 mg daily for three days and ciprofloxacin 500 mg PO daily for two days). His medical condition improved (Figure 5), and he was downgraded to the floor after nine days of ICU care. He was eventually discharged on April 11, 2020, with a prescription of 14 days of ciprofloxacin and instructions on social distancing and follow-up appointments.

**Table 3:** Case 2 arterial blood gas from March 28 to April 1, 2020 pCO_2_: partial pressure of carbon dioxide; pO_2_: partial pressure of oxygen; FiO_2_: fraction of inspired oxygen; P/F: PaO_2_/FiO_2_

Parameters	March 28, 2020	March 29, 2020	April 1, 2020
PH	7.326	7.266	7.341
pCO_2_	45.5	55.5	54.1
pO_2_	69.5	57.7	79
O_2_ saturation	91.2	83.6	94.1
O_2_ delivery	Venturi mask	Venturi mask	Venturi mask
FiO_2_	35%	80%	40%
P/F ratio	198	70	197

Case 3

**Figure 6:**
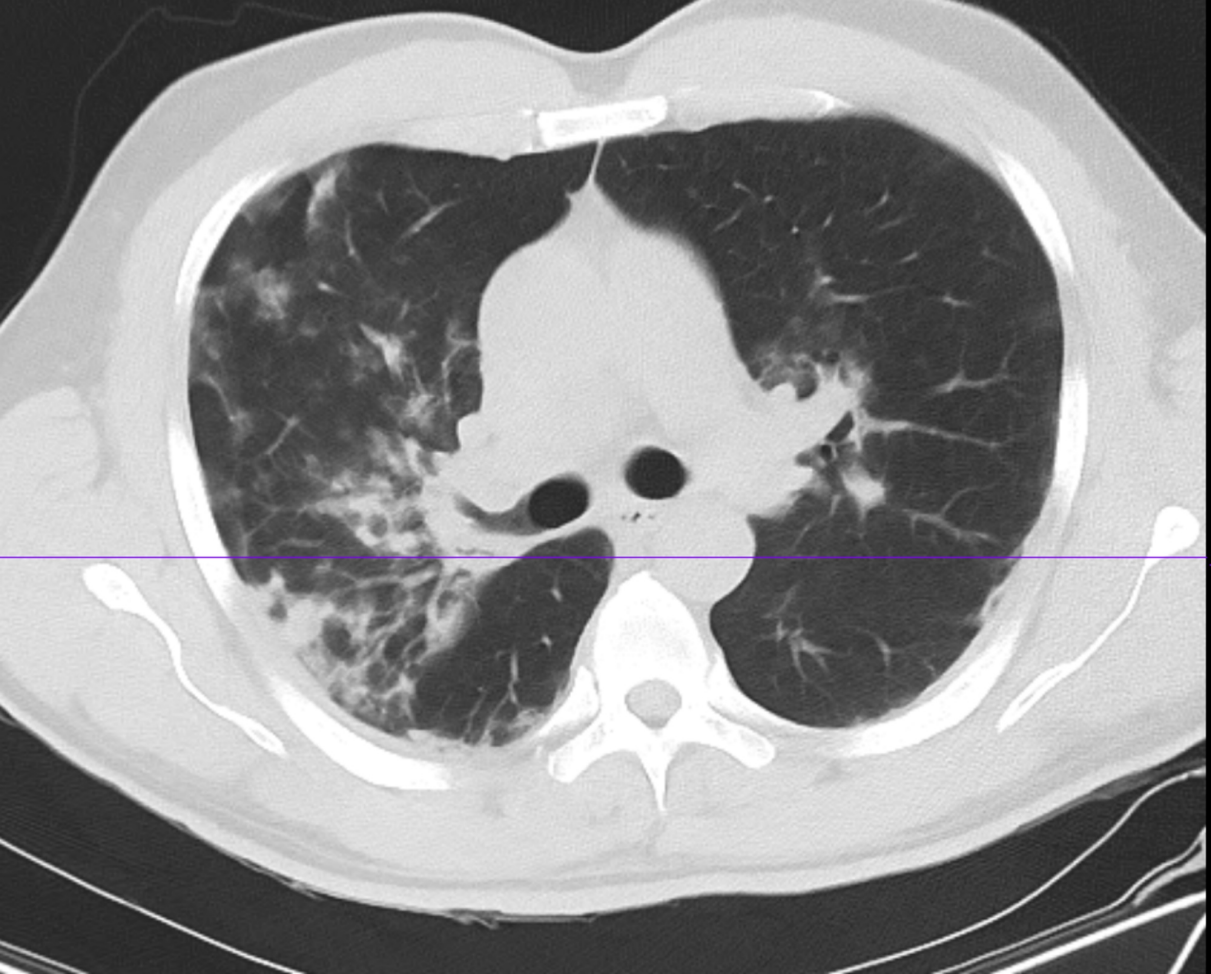
Case 3 chest CT on March 27, 2020 CT: computed tomography

**Figure 7:**
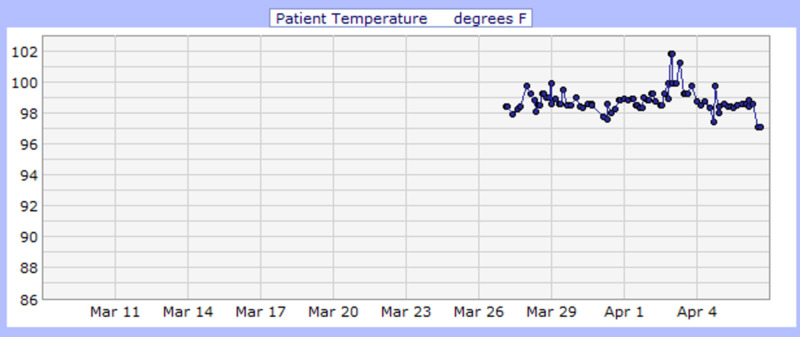
Case 3 temperature (°F)

**Figure 8:**
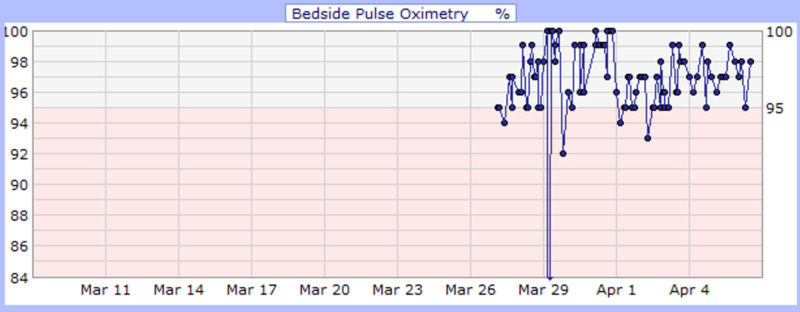
Case 3 bedside pulse oximetry (%)

On March 27, 2020, a 53-year-old male with a past medical history of HIV on anti-retroviral medications [abacavir/dolutegravir/lamivudine (Triumeq® tablet, ViiV Healthcare Limited, The Research Triangle, NC)] presented for feeling unwell for three days, specifically complaining of headaches, myalgia, dry cough, shortness of breath, and fever. His chest CT scan showed patchy infiltrates throughout both lungs with a ground-glass pattern, more prominent at the periphery and at the lung bases (Figure 6). Additionally, a COVID-19 RT-PCR test was positive, and two blood cultures showed no growth. Moreover, the electrocardiogram showed sinus rhythm with a corrected QT interval (QTc) of 418 ms. Other blood test results are shown in Table 1 (column: case 3). Overall, he finished a course of ceftriaxone (1 gm daily for six days), Tamiflu® (Roche Pharma, Basel, Switzerland, 75 mg daily for 10 days), hydroxychloroquine (400 mg for five days), and azithromycin (500 mg daily for four days) while QTc interval and electrolytes level were monitored. During his admission, he had spikes of fever; consequently, he was put on vancomycin and meropenem until he was afebrile for 72 hours. Furthermore, He had a few episodes of the mild drop in his oxygen saturation for which he was put on 2 L nasal cannula, and his saturation subsequently improved (Table 4). His symptoms improved gradually (Figures 7, 8), and he was discharged on April 6, 2020, and advised to practice social distancing.

**Table 4:** Case 3 arterial blood gas on March 27, 2020 pCO_2_: partial pressure of carbon dioxide; pO_2_: partial pressure of oxygen; FiO_2_: fraction of inspired oxygen; P/F: PaO_2_/FiO_2_

Parameters	March 27, 2020
PH	7.456
pCO_2_	42.7
pO_2_	59.5
O_2_ saturation	90%
O_2_ delivery	Room air
FiO_2_	21%
P/F ratio	283

## Discussion

The leading cause of mortality in COVID-19 patients is acute respiratory distress syndrome (ARDS). Some COVID-19 patients present with a cytokine profile resembling secondary haemophagocytic lymphohistiocytosis (sHLH), a fulminant and fatal hypercytokinemia with multiorgan failure, which is most commonly triggered by a viral infection and most frequently presents with fever, cytopenias, and hyperferritinemia with the involvement of the lungs (ARDS) [4-5]. Systemic elevation in pyrogenic cytokines such as interleukin-6 (IL-6), IL-10, and tumor necrosis factor-α (TNF-α) has been observed, suggesting that COVID-19 triggers hyper inflammation that initiates ARDS via a complex inter-relationship of cytokines and pro-inflammatory mediators involving humoral and cellular responses [3,4,6].

Data suggest that the host immune status may influence the progression of COVID-19. Some studies have shown higher levels of type I interferon (IFN-I) in HIV patients, which may help clear the COVID-19 infection. Additionally, delayed antibody response to COVID-19 in HIV patients has been reported, suggesting a possible influence of immune deficiency in HIV patients on clearance of COVID-19 [7].

The impact of multiple anti-retroviral drugs such as lopinavir-boosted ritonavir, darunavir, and remdesivir on COVID-19 has been studied. A case report by Holshue et al. showed clinical improvement in the first case of COVID-19 in the United States after the use of remdesivir [8]. Currently, Gilead Sciences has initiated two phase-3 studies investigating the efficacy of remdesivir for the treatment of COVID-19 [9]. Clinical trials have shown varying levels of effectiveness of lopinavir-boosted ritonavir on reducing mortality rates in COVID-19 patients. Some reports suggest that the addition of lopinavir/ritonavir to the initial treatment reduces the overall death rate and intubation rate [10,11]. Additionally, Janssen has announced that even though anecdotal instances of darunavir being used for the treatment of COVID-19 have been reported, it is ineffective in treating COVID-19 patients due to its low affinity to coronavirus protease [11-12].

## Conclusions

We propose that HIV could have modulated the immune system in these three patients in a way that led to a less severe immune response to COVID-19. Furthermore, the lower severity of COVID-19 in these HIV patients could be explained by the effect of the anti-retroviral medications these patients were taking. In order to confirm the validity of these hypotheses and show that it was not just the heterogenicity playing a role in such less severe immune response to COVID-19, extensive retrospective studies or prospective randomized control studies should be conducted to further evaluate these hypotheses. Additionally, we recommend further studies on anti-retroviral medications and their effect on COVID-19 patients.
